# Knockdown of ZFR suppresses cell proliferation and invasion of human pancreatic cancer

**DOI:** 10.1186/s40659-016-0086-3

**Published:** 2016-05-13

**Authors:** Xiaolan Zhao, Man Chen, Jishan Tan

**Affiliations:** Health Management Center, The First Affiliated Hospital of Third Military Medical University, NO. 30 Gaotanyan Street, Shapingba District, Chongqing, 400038 China; School of Laboratory Medicine, Chengdu Medical College, Chengdu, 610083 China; Department of Laboratory Medicine, Chengdu Military General Hospital, Chengdu, 610083 China

**Keywords:** Pancreatic cancer, RNA interference, Targeted therapy, Zinc finger RNA binding protein

## Abstract

**Background:**

Zinc finger RNA binding protein (ZFR) is involved in the regulation of growth and cancer development. However, little is known about ZFR function in pancreatic cancer.

**Methods:**

Herein, to investigate whether ZFR is involved in tumor growth, Oncomine microarray data was firstly used to evaluate ZFR gene expression in human pancreatic tumors. Then short hairpin RNA (shRNA) targeting ZFR was designed and delivered into PANC-1 pancreatic cancer cells to knock down ZFR expression. Cell viability, cell proliferation and cell cycle analysis after ZFR knockdown were determined by MTT, colony forming and FACS, respectively. In addition, cell migration and invasion were assessed using the Transwell system.

**Results:**

The expression of ZFR was significantly higher in pancreatic tumors than normal pancreas tissues by Oncomine database analysis. Knockdown of ZFR by shRNA-expressing lentivirus significantly decreased the viability and invasion ability of pancreatic cancer cells. Moreover, FACS analysis showed that knockdown of ZFR in PANC-1 cells caused a significant cell cycle arrest at G0/G1 phase. Furthermore, knockdown of ZFR decreased the levels of CDK2, CDK4, CyclinA and CyclinD1 and enhanced the expression of p27, which has evidenced by qRT-PCR and Western blot analysis.

**Conclusions:**

Knockdown of ZFR might provide a novel alternative to targeted therapy of pancreatic cancer and deserves further investigation.

## Background

Pancreatic cancer is currently the fourth most common cause of cancer-related mortality in USA [1], characterized by difficult diagnosis, distant metastasis and aggressive local invasion at an early stage [2]. The five-year survival rate among patients with pancreatic cancer is less than 5 % [3, 4] and almost all the patients succumbed to their disease within 2 years [5]. In the last two decades, strategies including surgery, radiation and chemotherapy, have failed to improve long-term survival [6]. Recent efforts have focused on the application of novel targeted agents based on a better understanding of the mechanisms involved in tumor progression.

Zinc finger proteins, encoded by zinc finger RNA binding (ZFR), has been reported to play an important role in DNA binding and regulate growth and development [7]. ZFR is an ancient and highly conserved chromosome-associated protein from nematodes to mammals [8]. The murine ZFR protein was identified in a screen for RNA-binding proteins expressed during spermatogenesis [9]. The human ZFR appears to be involved in the regulation of alternative pre-mRNA splicing [10] and was identified in a screen for factors that interact with the pre-mRNA splicing activator RNPS1 [11]. Transcript levels were determined for ZFR in various human tissues but undetectable in skeletal muscle [10]. Knockdown expression of ZFR through RNA interference in neurons relocated specifically the Staufen 2 (62), isoform to the nucleus, which suggest that ZFR is critical for Staufen 2 isoform specific nucleocytoplasmic shuttling in neurons and likely acts key function during early steps of RNA transport and localization [12]. TDP-43 was shown to be a major disease protein in most cases of amyotrophic lateral sclerosis (ALS), *tau*-negative frontotemporal lobar degeneration (FTLD), and inclusion body myopathy. Mass spectrometry identified ZFR as one of the TDP-43 interacting proteins [13]. In addition, ZFR has been demonstrated to direct the targeted chromosomal integration of a plasmid at specific recognition sites distributed throughout human genomes in several cell types [14]. Despite recent obtained advances in understanding the biology function of ZFR, there is poor report about the effect of ZFR on pancreatic cancer diagnosis and prognosis.

In the present study, we aimed to investigate whether ZFR functions as potential target in pancreatic cancer, one stable knockdown of ZFR cell line model was constructed using lentivirus-mediated RNA interference technique. Based on constructed ZFR silencing cell model, we further determined the biological function of ZFR silencing on pancreatic cancer as well as downstream target proteins expression level.

## Methods

### Materials

Dulbecco’s modified Eagle’s medium (DMEM) was obtained from Hyclone (Catalog Number: SH30243.01B+, Logan, Utah, USA). Fetal bovine serum (FBS) was obtained from Biowest (Catalog Number: S1810, Loire Valley, France). The lentiviral expression vector (pFH-L) and packaging vectors (pVSVG-I and pCMVΔR8.92) were purchased from Hollybio (Shanghai, China). Lipofectamine 2000 and TRIzol^®^ Reagent was purchased from Invitrogen (Carlsbad, CA, USA). M-MLV Reverse transcriptase was purchased from Promega (Madison, WI, USA). All other chemicals were obtained from Sigma-Aldrich (St. Louis, MO, USA).

### Analysis of oncomine data

To determine the expression pattern of ZFR in pancreatic cancer, two datasets, including Badea Pancreas [15] and Segara Pancreas [16] in Oncomine database (www.oncomine.org) were used. The gene expression of ZFR was compared between pancreatic cancer tissues with normal pancreatic tissues according to the standard procedures as previously described [17].

### Cell culture

Human pancreatic cancer cell line, PANC-1 and human embryonic kidney cell line 293T were obtained from the Cell Bank of Chinese Academy of Science (Shanghai, China). Both cell lines were cultured in DMEM supplemented with 10 % FBS at 37 °C with 5 % CO_2_.

### Construction of recombinant lentiviral vector

The cDNA sequence of ZFR was obtained from Gen Bank (NM_016107). The shRNA target sequence for ZFR was 5′-GCCAAGGTGCAACTCAGTATACTCGAGTATACTGAGTTGCACCTTGGCTTTTT-3′, which was subjected to BLAST analysis against the human genome database to eliminate cross-silence phenomena with nontarget genes. A scrambled fragment (5′-GCGGAGGGTTTGAAAGAATATCTCGAGATATTCTTTCAAACCCTCCGCTTTTTT-3′) that has no significant homology with human gene sequences was used as a negative control. DNA oligonucleotides to produce plasmid-based shRNA were cloned into the pFH-L vector by use of *Nhe*I/*Pac*I restriction sites. The lentiviral expression vector (pFH-L) and packaging vectors (pVSVG-I and pCMVΔR8.92) were cotransfected into 293T cells with Lipofectamine 2000 according to the manufacturer’s instructions. The supernatant was collected 48 h later, centrifuged (4000*g*, 4 °C, 10 min) to remove cell debris, filtered through 0.45-μm cellulose acetate filters, and then concentrated again (4000*g*, 4 °C, 15 min). The lentiviral vectors expressed green fluorescence protein (GFP), which allowed for titering and measurement of their infection efficiency in transduced cells. PANC-1 cells were dispensed into 6-well plates at a density of 50,000 cells per well and transduced with shRNA-expressing lentivirus at a multiplicity of infection (MOI) of 75. GFP expression was observed by fluorescent microscopy 4 days post-transduction.

### Quantitative real-time polymerase chain reaction (qRT-PCR)

Cells transduced with shRNA-expressing lentivirus were divided into two groups (shCon, shZFR). PANC-1 cells were harvested after lentivirus transduction for 6 days. Total cellular RNA was extracted using Trizol reagent and reversely transcripted to cDNA by M-MLV reverse transcriptase according to the manufacturer’s instructions. qRT-PCR products were detected with SYBR Green on BioRad Connect Real-Time PCR platform following the procedure: denaturation at 95 °C for 1 min, 40 cycles of denaturation at 95 °C for 5 s and extension at 60 °C for 20 s. Specific cDNAs were then amplified by qRT-PCR using the following primers: ZFR, 5′-TCCCAATGCTAAGGAGATGC-3′ (forward) and 5′-TTCTTCTCGTCTTCGCCAGT-3′ (reverse); CDK2, 5′-TCCAGGATGTGACCAAGCC-3′ (forward) and 5′-CTGAGTCCAAATAGCCCAAGG-3′ (reverse); CyclinA, 5′-GTTCCTCCTTGGAAAGCAAAC-3′ (forward), 5′-GGGCATCTTCACGCTCTATTT-3′ (reverse); CyclinD1, 5′-GCCCTCGGTGTCCTACTTC-3′ (forward), 5′-CCTCCTCGCACTTCTGTTCC-3′ (reverse); p27, 5′-GCTACCCTTGACAAGAAAAGAC-3′ (forward), 5′-AAAGTATGCTTACTAAAGGTCCTG-3′ (reverse) and β-actin, 5′-GTGGACATCCGCAAAGAC-3′ (forward) and 5′-AAAGGGTGTAACGCAACTA-3′ (reverse). The β-actin was used as internal control. Relative quantitation was analyzed by taking the difference ΔC(T) between the C(T) of β-actin and C(T) of ZFR and computing 2-ΔΔC(T) as described previously [18].

### Western blot analysis

Cells transduced with shRNA-expressing lentivirus were divided into two groups (shCon, shZFR). PANC-1 cells were harvested after lentivirus transduction for 6 days. Total protein was extracted with 2 × SDS Sample Buffer [100 mM Tris–HCl (pH 6.8), 10 mM EDTA, 4 % SDS, 10 % Glycine]. Total protein concentration was determined by BCA assay. Protein extracts were separated by 10 % SDS-PAGE and transferred to PVDF membranes. The membranes were blocked with 5 % nonfat dry milk in Tris-buffered saline with Tween 20 (TBST) for 1 h at 37 °C, and then incubated overnight at 4 °C in TBST with primary antibody, including rabbit anti-ZFR (1:1000, SAB2104153, sigma), mouse anti-CDK4 (1:500, #2906, cell signaling), rabbit anti-CDK2 (1:1000, #2546, cell signaling) and rabbit anti-GAPDH (1:100000, 10494-1-AP, Santa cruz). Following incubation with horseradish peroxidase-conjugated goat anti-rabbit/mouse secondary antibody for 1 h, the membranes were detected using enhanced chemiluminescence (ECL) kit (Amersham) and visualized by exposure to X-ray film. GAPDH was used as a control to verify equal protein loading.

### MTT assay

To detect cell viability, 3-(4, 5-dimethylthiazol-2-yl)-2, 5-diphenyl-tetrazolium bromide (MTT) colorimetric assay was performed 4 days after lentivirus transduction. Briefly, PANC-1 cells were dispensed into 96-well plates at a density of 2000 per well. The plates were incubated for one to 5 days at 37 °C. On each day, 100 ml of MTT (5 mg/ml) was added and incubated for 4 h. Afterwards, the entire supernatant was discarded and acidic isopropanol (10 % SDS, 5 % isopropanol and 0.01 mol/L HCl) was added at a volume of 100 μl per well and incubated at 37 °C for 10 min. The absorbance at 595 nm of each well was determined using an ELISA reader.

### Colony formation assay

To detect cell proliferation, colony formation assay was performed 4 days after lentivirus transduction. Briefly, PANC-1 cells were dispensed into 6-well plates at a density of 500 per well. The culture medium was changed at three-day intervals. PANC-1 cells were cultured for 10 days until the most single colony contains more than 50 cells. The colonies were stained with crystal violet for 15 min, and then washed with water and air-dried. Cell colonies were captured and counted under a microscope.

### Fluorescence-activated cell sorting analysis (FACS)

To detect cell cycle progression, flow cytometry assay was performed 7 days after lentivirus transduction as described previously [19]. Briefly, PANC-1 cells were dispensed into 6-cm dishes at a density of 200,000 per dish. After culture at 37 °C for 40 h, cells were harvested, fixed in 70 % ethanol, and stored overnight at 4 °C. The cells were then treated with NaCl/Pi staining solution (50 µg/mL PI and 100 µg/mL RNase A). Following incubation for 1 h in the dark at room temperature, cells were analyzed by flow cytometry (FACSCalibur; Becton–Dickinson, San Jose, CA, USA). The fractions of the cells in G0/G1, S and G2/M phases were analyzed with dedicated software (Becton–Dickinson).

### Cell migration and invasion assays

The migration and invasion ability of PANC-1 following ZFR knockdown were evaluated using Transwell (8 µm pore size, Millipore). Briefly, cells were suspended in serum-free medium and seeded into Transwell inserts either uncoated (for migration assay) or coated (for invasion assay) with growth factor-reduced Matrigel (BD Biosciences, Bedford, MA). Botton wells were filled with complete medium. Then the invaded cells were fixed with methanol and stained with a crystal violet solution. Finally, the number of migratory and invasive cells were counted in five fandom fields under a microscope at 200 × magnification.

### Statistical analysis

Statistical analysis was performed using SPSS 13.0 software package (SPSS Inc, Chicago, IL, USA). Data were expressed as mean ± standard deviation from three independent experiments. Statistical differences between the groups were compared using Student’s *t* test. A P value of less than 0.05 was considered statistically significant.

## Results

### ZFR is upregulated in pancreatic cancer

**Fig. 1:**
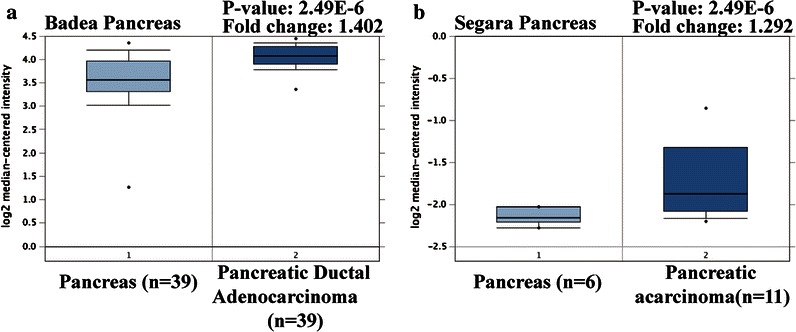
Oncomine studies analysis. The expression of ZFR in pancreatic tumors than pancreas tissues of Badea Pancreas (**a**) and Segara Pancreas (**b**)

ZFR mRNA levels in human pancreatic cancer tissues were investigated using two datasets from the publicly available oncomine database. As shown in Fig. 1, all of the two datasets showed a significantly higher level of ZFR expression in pancreatic cancer tissues compared with the normal pancreatic tissues (*p* < 0.01).

### Knockdown of endogenous ZFR by shRNA-expressing lentivirus

**Fig. 2:**
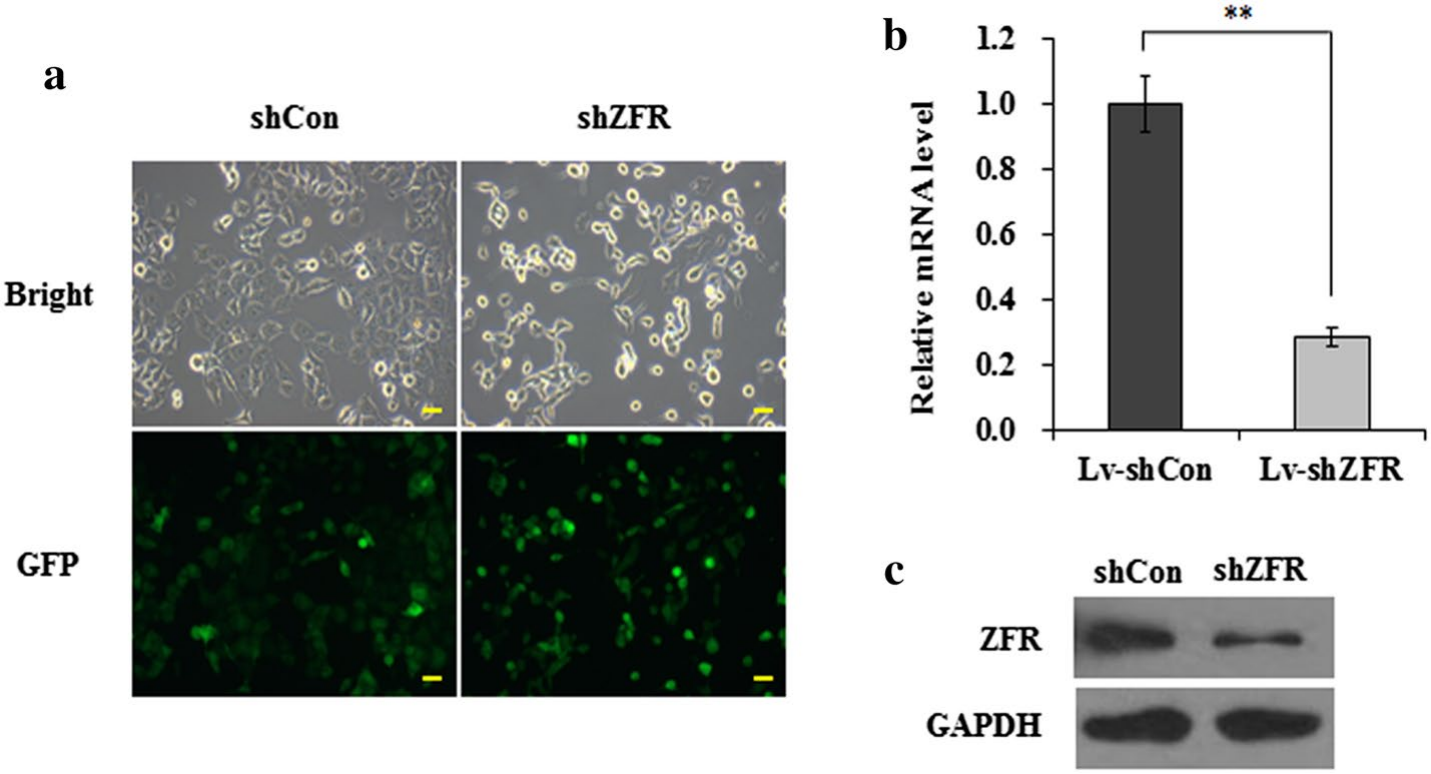
Lentivirus stably expressed shRNA targeting ZFR in PANC-1 cells. **a** Evaluation of the lentivirus transduction rate, which was more than 80 % as calculated by cellular enumeration using fluorescence (*GFP* green fluorescence protein) and light microscopy (*Bright*). **b** Quantitative analysis of ZFR knockdown efficiency in PANC-1 pancreatic cancer cells assessed by qPCR (β-actin gene was used as an internal control). **c** Representative western blot showing ZFR knockdown efficiency determined in PANC-1 pancreatic cancer cells. GAPDH protein was used as an internal control. Data are mean ± S.D. (n = 3; *t*-test). **p < 0.01; *scale bar* 100 μm. *shCon* short hairpin control RNA; *sh ZFR* short hairpin Zinc finger RNA

PANC-1 cells were transduced with shRNA-expressing lentivirus (shCon: control group or shZFR: short hairpin Zinc finger RNA), respectively. The infection efficiency was confirmed by evaluating GFP expression levels. As depicted in Fig. 2a, over 80 % of cells infected with shZFR expressed GFP, which suggested that the lentivirus transduction was successful and highly efficient. The effect of shZFR on ZFR expression in PANC-1 cells was investigated by qPCR and western blot analysis. As depicted in Fig. 2b, the mRNA levels of ZFR was significantly down regulated in cells infected with shZFR than in cells infected with shCon (*p* < 0.01). What’s more, protein expression levels of ZFR were also obviously down regulated in cells infected with shZFR than in cells infected with shCon. There results suggested shZFR could specifically and strongly suppress expression of endogenous ZFR in pancreatic cancer cells.

### Knockdown of ZFR impairs cell viability and colony formation

**Fig. 3:**
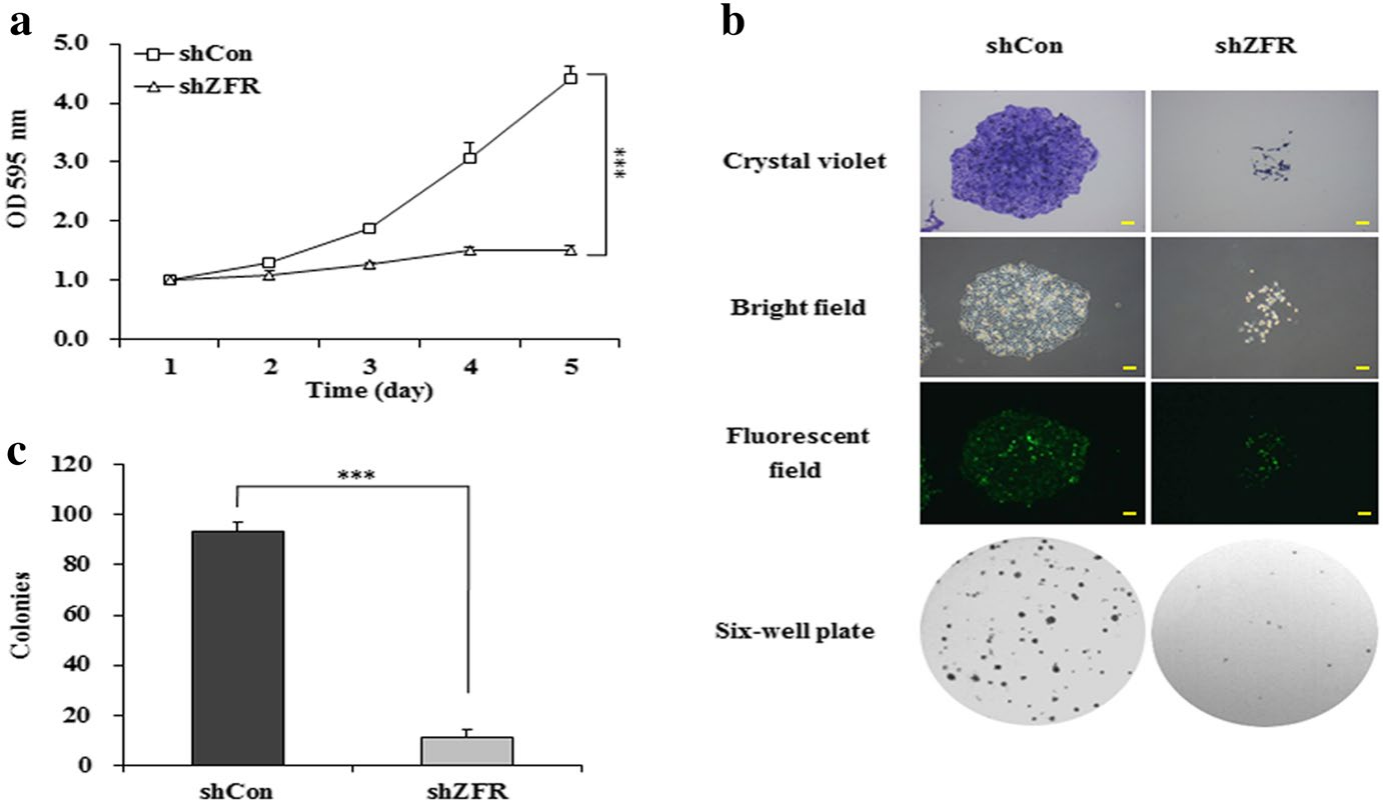
Depletion of ZFR decreases the viability and proliferation of pancreatic cancer cells. **a** MTT showing growth curves determined in PANC-1 cells. The number of viable cells was fewer in cells infected with shZFR than in cells infected with shCon. **b** Representative colony formation showing clonogenic survival determined in PANC-1 cells. **c** The number of colonies was fewer in cells infected with shZFR than in cells infected with shCon. Data are mean ± S.D. (n = 3; t-test). ***p < 0.001; *scale bar* 250 μm. *shCon* short hairpin control RNA; *sh ZFR* short hairpin Zinc finger RNA

After infection by ZFR shRNA-expressing lentivirus for 4 days, we investigated the cell viability for five consecutive days by MTT assay. On day 4, compared with shCon, the number of viable cells infected with shZFR was reduced by 51 % (*p* < 0.001, Fig. 3a). The reduction in growth curve of cells infected with shZFR was more obvious on day 5, which indicates that shZFR could strongly decrease the viability of PANC-1 cells.

In addition, colony formation assay was performed to determine cell proliferation in vitro. Consistently, the colony formation was significantly disrupted in cells infected with shZFR. As shown in Fig. 3b, the size of single colony in cells infected with shZFR was much smaller than that in cell infected with shCon, and the total number of colonies in 6-well plates was remarkably decreased in PANC-1 cells transduced with shZFR, in comparison with the control. Specifically, there were average 93 colonies in control group, while only average 11 colonies in shZFR treatment group (*p* < 0.001, Fig. 3c). These results indicate that shZFR could efficiently inhibit cell viability and proliferation of pancreatic cancer cells.

### Knockdown of ZFR causes cell cycle arrest

**Fig. 4:**
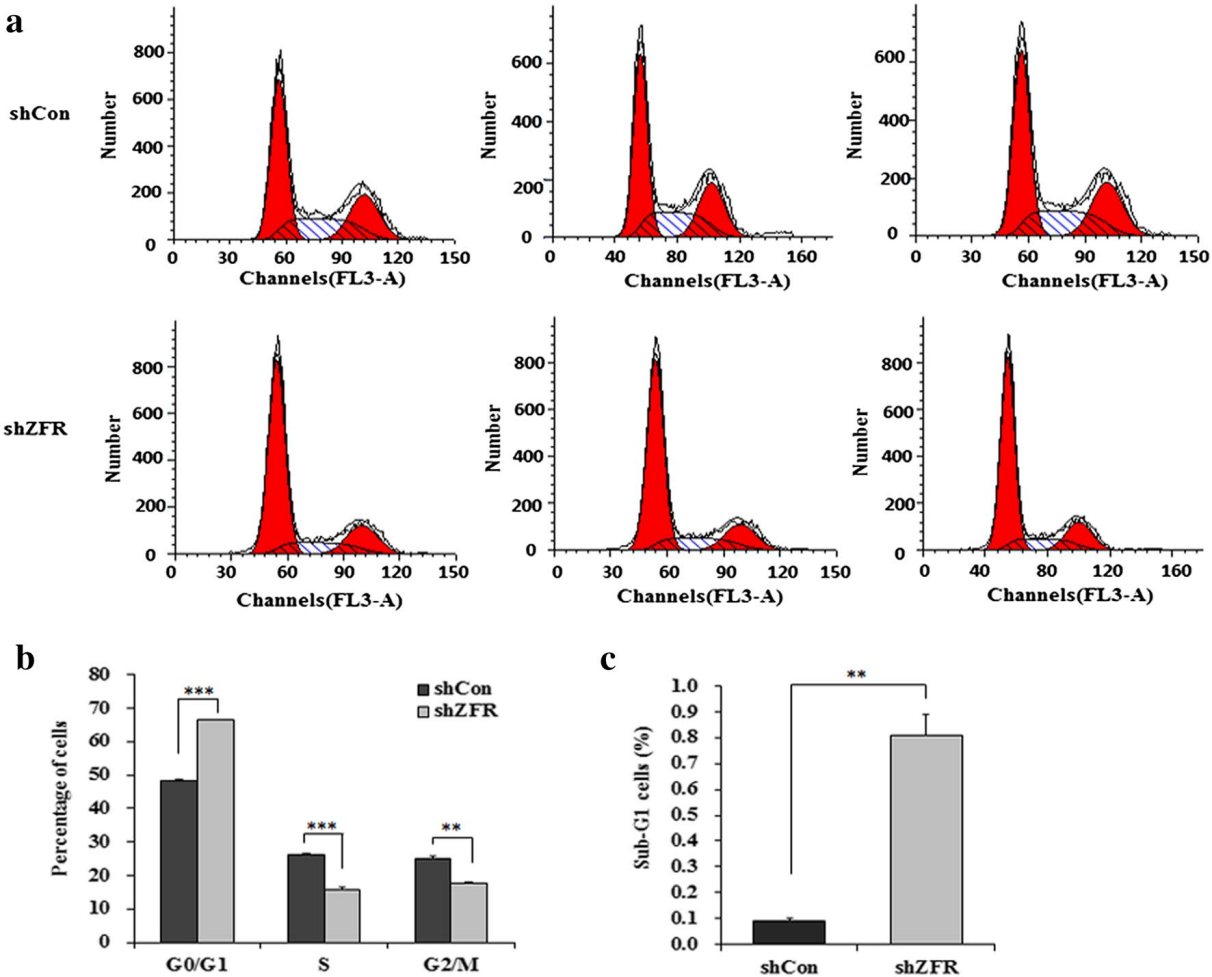
Depletion of ZFR arrests cell cycle progression in pancreatic cancer cells. **a** Comparison of the cell population in G0/G1, S and G2/M phase between shCon and shZFR groups, as assessed by flow cytometry. **b** The percentage of cells in G0/G1 phase was significantly higher in cells infected with shZFR than in cells infected with shCon, while the percentages of cells in S and G2/M phases were simultaneously reduced. **c** The percentage of cells in sub-G1 phase was significantly higher in cells infected with shZFR than in cells infected with shCon. Data are mean ± S.D. (n = 3; t-test). **p < 0.01; ***p < 0.001. *shCon* short hairpin control RNA; *sh ZFR* short hairpin Zinc finger RNA

Flow cytometry assay was used to determine the effect of shZFR on the cell cycle of PANC-1 cells. Representative images of cell cycle distribution were presented as Fig. 4a. As depicted in Fig. 4b, the cell percentage of G0/G1 phase was increased from 48.42 % in cells infected with shCon to 66.56 % in cells infected with shZFR. Meanwhile, the cell percentage of S phase was decreased from 26.31 % in control group to 15.91 % in shZFR treatment group, and the cell percentage of G2/M phase was decreased from 25.21 to 17.53 %. These results indicate that shZFR could strongly arrest PANC-1 cells in the G0/G1 phase of the cell cycle (*p* < 0.001). Moreover, the cell percentage of sub-G1 phase, representing cell early apoptosis, was increased from 0.09 % in control group to 0.81 % in shZFR treatment group (*p* < 0.001, Fig. 4c). Taken together, these results suggest knockdown of ZFR could inhibit pancreatic cancer cell proliferation might via inducing G0/G1 cell cycle arrest.

### Knockdown of ZFR altered cell cycle-associated proteins in PANC-1 cells

**Fig. 5:**
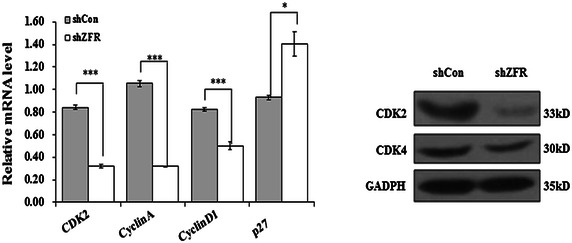
Depletion of ZFR suppresses cell cycle-associated proteins in pancreatic cancer cells. Western blot analysis of G0/G1 phase-associated proteins in PANC-1 cells after shZFR infection. GAPDH protein was used as an internal control. *shCon* short hairpin control RNA; *sh ZFR* short hairpin Zinc finger RNA

To illuminate the molecular basis for cell cycle arrest caused by ZFR knockdown, the expression levels of cell cycle regulatory molecular were analyzed by qRT-PCR and Western blot analysis. As shown in Fig. 5, knockdown of ZFR significantly suppressed the expression levels of CDK2, CDK4, Cyclin A and CyclinD1 and enhanced the expression levels of p27 in PANC-1 cells. These results suggest that knockdown of ZFR in PANC-1 cells leads to cell cycle arrest at G0/G1 phase probably via alteration of cell cycle regulatory molecular.

### Knockdown of ZFR suppressed cell migration and invasion ability in PANC-1 cells

**Fig. 6:**
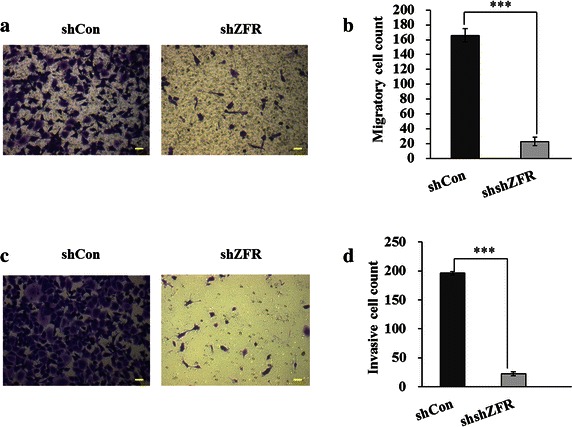
Depletion of ZFR inhibited cell migratory and invasive ability in pancreatic cancer cells. **a** Representative images of migratory cells stained with *crystal violet*. **b** Quantitative analysis of migratory cell in PANC-1 cells following ZFR knockdown. **c** Representative images of invasive cells stained with *crystal violet*. **d** Quantitative analysis of invasive cell in PANC-1 cells following ZFR knockdown. Mean ± standard deviation (SD) of three independent experiments. ***p < 0.001 compared with controls

What’s more, we investigated whether ZFR affected cell migration and invasion ability in pancreatic cancer. As shown in Fig. 6, suppression of ZFR significantly inhibited the migration and invasion of PANC-1 cells, as indicated by a marked decrease in the number of cells that invaded the bottom well (*p* < 0.001).

## Discussion

Despite progresses in diagnosis and treatment, pancreatic cancer is still considered as the worst prognosis of all solid malignant tumors, with a five-year survival of less than 5 % [20]. Therefore, a better understanding of the molecular mechanisms involved in the progression of pancreatic cancer is particularly important for improving the treatment efficacy for the pancreatic cancer patients. RNA-binding proteins containing the double-stranded RNA-binding domain (dsRBD, RBD, or DZF) represent a variety of proteins with diverse cellular functions [21]. ZFR is a conserved protein with three copies of the C_2_H_2_ zinc finger motif in the N-terminal region and a C-terminal DZF flanked by two nuclear localization signals (NLSs). ZFR can be recognized by nuclear factor NF45 through the DZF-domain, revealing that ZFR may form a variety of cellular complexes with other DZF-domain proteins, which are involved in a variety of cellular processes [22]. Despite most of these proteins are involved in the regulation of gene expression, the finger structure and functions are not well known.

In this study, we provide new evidence demonstrating that ZFR may be a potential tumor marker in human pancreatic cancer. ZFR was firstly found to be significantly highly expressed in pancreatic tumor tissues compared with normal pancreatic tissues by Oncomine database analysis. Then we designed shRNAs to specifically block its endogenous expression in human pancreatic cell line PANC-1 cells. Functional analysis showed knockdown expression of endogenous ZFR significantly decreased the viability and invasion ability of PANC-1 cells. Moreover, FACS analysis showed that knockdown expression of ZFR induced a significant cell cycle arrest in the G0/G1 phase. Cell proliferation proceeds as an orderly progression through the cell cycle, which is governed by protein complexes composed of cyclins and cyclin-dependent kinases (cdks) [23]. Besides, CDKs are tightly regulated by the association with cyclins. Cyclins and CDKs are two kinds of crucial regulatory molecules determining cell cycle progression [24]. CDK2, CDK4 and CDK6 are activated in association with the D-type Cyclins or Cyclin E during G1 progression and G1-S transition [25]. Moreover, sequential activation of the cyclin-CDK complexes contributes to the uncontrolled growth which characterizes cancer. [26]. Herein, we found G0/G1 phase arrest by ZFR silencing in PANC-1 cells and compared with that in shCon, the expression of CDK2 and CDK4 decreased in shZFR infected cells, which suggests knockdown of ZFR induces cell arrest through the inhibition of Cyclins and CDKs.

Tristetraprolin (TTP or ZFP36), a tandem CCCH zinc finger RNA binding protein, has been shown to regulate an important subset of cancer-related genes that are involved in breast cancer cell growth, invasion and metastasis in a 3′UTR- and ARE-dependent manner [27]. Therefore, it is likely that ZFR could modulate pancreatic cancer growth via the regulation of a subset of cancer-related genes involved in cellular growth and apoptosis. Further investigations are needed to reveal the molecular mechanism of the oncogenic function of ZFR in pancreatic cancer cells, which would be helpful for early detection or diagnosis of pancreatic cancer.

In summary, knockdown expression of ZFR by RNAi significantly inhibits the growth of PANC-1 cells by inducing cell cycle arrest. Our findings provide new evidence that ZFR plays an essential role in cell growth and may be a potent therapeutic target in human pancreatic cancer, based on which we could develop more effective diagnosis approaches for pancreatic cancer to prong the patients survival.
