# Expression of Concern to: Knockdown of ZFR suppresses cell proliferation and invasion of human pancreatic cancer

**DOI:** 10.1186/s40659-018-0171-x

**Published:** 2018-06-22

**Authors:** Xiaolan Zhao, Man Chen, Jishan Tan

**Affiliations:** 1 Health Management Center, The First Affiliated Hospital of Third Military Medical University, No 30 Gaotanyan Street, Shapingba District, Chongqing, 400038 China; 2 School of Laboratory Medicine, Chengdu Medical College, Chengdu, 610083 China; 3 Department of Laboratory Medicine, Chengdu Military General Hospital, Chengdu, 610083 China

## Expression of Concern: Biol Res (2016) 49:26 10.1186/s40659-016-0086-3

Concerns have been raised about this article [1] relating to the appropriateness of the use of the shRNA (5′-GCGGAGGGTTTGAAAGAATATCTCGAGATATTCTTTCAAACCCTCCGCTTTTTT-3′) as a non-targeting control and similarities in text and formatting with other published articles. This is currently under investigation and appropriate editorial action will be taken once the investigation is concluded. The authors did not respond to our correspondence regarding this expression of concern.
